# On the Preparation of Nourishing Broths from Fresh and Dry Bones

**Published:** 1804-10-01

**Authors:** 


### *On the Preparation of nourishing Broths from fresh and dry Bones, by* Mr. HERMBSTAEDT.

FLESH, without fat and bones, consists of jelly, a particular odorous substance, a fibrous substance, and much water; all which constituents are met with in the flesh of every animal, and nearly of the same quality; but with respect to their quantity, the different sorts of flesh greatly differ from each other. I have made my experiments on beef, veal, mutton, and pork; the results of which are, that the constituents were found in these sorts of flesh, on an average, to be in one pound (16 ounces Germ. civil weight)

| | | | |
|---|---|---|---|
| 1. Dry, nourishing jelly | 2 | to | 2 $\frac{1}{2}$ ounces |
| 2. Fat - - - - - - - | $\frac{2}{10}$ | | $\frac{1}{4}$ |
| 3. Fibrous substance - | 2 | | 2 $\frac{1}{2}$ |
| 4. Watery particles - | 11 $\frac{8}{10}$ | | 10 $\frac{1}{4}$ |
| | 16 | | 16 |

Of

Of these constituents, the jelly which is extracted by water may be considered as the nutritive matter, the fibrous substance merely satiating the stomach; for which purpose, other substances may be employed. Bones, in their fresh state, freed from flesh, fat, and membranes, contain in one pound the following constituents.

| | | | |
|---|---|---|---|
| 1. Dry nourishing jelly and odorous matter | 4 | to | $4\frac{1}{2}$ oz. |
| 2. Fat - - - - - - - - - - - - - - - | 2 | | $2\frac{1}{2}$ |
| 3. Bony substance - - - - - - - - - | 8 | | $7\frac{1}{2}$ |
| 4. Watery particles - - - - - - - - - | 2 | | $2\frac{1}{2}$ |
| | — | | — |

The jelly of bones perfectly resembles that of flesh, and the bony substance is very analogous to the fibrous substance of flesh; consequently, the bones seem merely to be an indurated flesh. Both parts differ from other animal substances by containing a peculiar odorous matter, from which the agreeable and refreshing smell of broth and roasted meat arises, and of which horn, the tendinous parts, the membranes and intestines are entirely deprived; and the jelly which may be procured from these substances is not so agreeable as that obtained from flesh and bones, but rather resembles glue. The jelly, the fat, and the odorous matter are separated by boiling flesh and bones in water, whereas the fibrous and bony matters remain undissolved in a tasteless state.

The decoctions of flesh and bones being cooled, the fat is separated on their surface, in a coagulable state, while the broth runs into a trembling jelly, capable of being cut into pieces. On evaporating this jelly to perfect dryness, it may be preserved many years, without danger of corruption; it readily dissolves in hot water, forming a liquid, very agreeable, and nourishing broth.

According to the above statement, one pound of fresh bones contains, on an average, double the quantity of dry nourishing jelly as one pound of fresh flesh without bones. But as flesh sold in the market contains at least 25lb. of bones in every 100 weight, which by the common boiling yields but a small portion of their virtues, the proportion of constituents in 1lb. of flesh with bones may be stated in the following manner.

| | | |
|---|---|---|
| 1. Bones - - - - - | 4 | ounces |
| 2. Jelly - - - - - | $1\frac{1}{2}$ | |
| 3. Fat - - - - - - | $\frac{3}{8}$ | |
| 4. Fibrous substance | $1\frac{1}{2}$ | |
| 5. Watery particles | $8\frac{1}{2}\frac{5}{8}$ | |
| | — | |

The proportion of jelly, therefore, extracted from flesh with bones, to that from bones only, is = 3 : 8 or = 1 : $2\frac{2}{3}$, consequently, each pound of bones is $2\frac{2}{3}$ times more value than one pound of flesh, if compared with respect to their nutritive virtues. Bones that have been boiled with the flesh in the common way, retain the greatest part of their jelly, and yield, according to my method, at least three-fourths as much jelly and fat as fresh bones which have not been boiled. The method of separating the nutritive particles from bones is not expensive; and although the price of fresh bones be equal to that of flesh, would, notwithstanding, be advantageous. But when the bones of boiled or roasted meat are employed, which may be purchased at a very low price, the fat alone that is obtained by my method of boiling, will defray the expence of fuel, &c. and the jelly itself scarcely cost any thing. Those bones that are usually thrown away might be advantageously employed in making food for the poor, as well as for hospitals; and they will likewise afford, with the necessary additions, wholesome and agreeable soups for families; by which means the expences attending the purchase of meat will be considerably reduced. Large public institutions for supporting poor and helpless people may also derive great advantages from this manner of preparing nourishing jellies. Now, supposing that one institution consumes twenty oxen per month, this will, in the course of a year, amount to two hundred and forty; and supposing that each ox weighs 500lb. the whole mass of beef consumed in this period of time will average 120,000 lb. which, at a low calculation, contains 25lb. of bones in every cwt. or 30,000 lb. According to my experiments, 1lb. of bones, in their fresh state, contains 4 ounces of dry jelly and 1 ounce of fat; consequently, those 30,000 lb. of bones contain 120,000 oz. or 7,500lb. of dry jelly, and 30,000 oz. or 1,875 lb. of fat. But as 1lb. of such jelly, considered as nutritive matter, is equal to 8 lb. of flesh, those 7,500 lb. of dry jelly from bones are of the same value as 60,000 lb. of flesh. Or, if we take the price of 1 lb. of beef to be equal to 2 groshen or about 3d. $\frac{1}{2}$, the value will be 5,000 rixdollars. And if we bring the fat obtained from the bones into computation, at 6d. each lb. those 18,75 lb. of fat will be worth 312 rixdollars; consequently, such an institution might yearly gain the sum of 5,312$\frac{1}{2}$ rixdollars; a diminution of expence which certainly deserves attention. The greatest advantage may arise from the introduction of such a jelly to to armies and field hospitals, particularly if it were prepared in great quantities, as it might be readily transported without any fear of corruption. Soldiers would always have a cheap, nourishing, and wholesome diet. Besieged places could in this manner more easily guard against the want of meat; and to the sick and wounded, it would afford a nutritive food. The jelly may be rendered more fit for preservation by salt, spices, onions, &c. and I again maintain, that there is not the least difference between the jelly of bones and that prepared from flesh.

Other advantages which the preparation of this jelly seems to promise, I shall more amply detail in a particular work which I intend to publish on this subject.

